# Software tools for implementing simulation studies in adaptive seamless designs: introducing R package ASD

**DOI:** 10.1186/1745-6215-12-S1-A8

**Published:** 2011-12-13

**Authors:** Nick Parsons, Tim Friede, Susan Todd, Nigel Stallard

**Affiliations:** 1 Warwick Medical School, The University of Warwick, Coventry, UK; 2 University Medical Center Göttingen, Göttingen, Germany; 3 Applied Statistics, University of Reading, Reading, UK

## 

Adaptive designs for clinical trials have the potential to improve the efficiency of clinical research by, for instance, seamlessly combining different stages of a clinical development programme. Adaptive seamless designs (ASD) combining phases II and III with adaptations such as treatment or subgroup selection are becoming increasingly widely used. The work required to develop application specific software for each new trial is a potential barrier to the more extensive use of ASD, particularly as simulation studies are often required at the planning stage to explore a range of options prior to deciding on an appropriate design. A number of common analysis methods (e.g. weighted inverse normal combination functions) and simulation tools for assessing the performance of a number of design options, are available in some widely used commercial packages, but currently no free software exists for planning and simulation of ASD. Parsons *et al. *[1] developed R package asd specifically for undertaking simulations to investigate designs for treatment selection based on early outcomes [2]. The freely available software simulates normal test statistics, rather than individual patient data, in the setting of treatment selection, providing flexibility to deal with combinations of scales (e.g. normal, binary) for both early and final outcomes, for two stage designs. Two examples for normally distributed and binomially distributed final outcomes are described with code demonstrating implementation in the software and output to show how this methodology can be used explore a range of options prior to deciding on an appropriate trial design. The software is flexible and fast, allowing many design options to be explored, and is currently in the process of being extended in a new version that will also include simulations for subpopulation selection. The software is available from the Comprehensive R Archive Network [http://cran.r-project.org/] and more details can be found at the Package Website [http://cran.r-project.org/web/packages/asd/].
